# A precise TIN clipping algorithm for the digital mining design of the opencast coal mine

**DOI:** 10.1371/journal.pone.0281864

**Published:** 2023-02-16

**Authors:** Jingchang Zhao, Xiangbo Wang, Dong Wang, Guangwei Liu

**Affiliations:** 1 Institute of Technology and Equipment for Exploitation and Utilization of Mineral Resources, Liaoning Technical University, Fuxin, Liaoning, China; 2 School of Mining, Liaoning Technical University, Fuxin, Liaoning, China; Bu-Ali Sina University: Bu Ali Sina University, ISLAMIC REPUBLIC OF IRAN

## Abstract

The triangulated irregular network (TIN) clipping algorithm is one of the vital algorithms for the digital mining design of opencast coal mines based on the geological digital elevation model (DEM) described by TIN. This paper gives a precise TIN clipping algorithm applied in the digital mining design of the opencast coal mine. To improve the algorithm’s efficiency, a spatial grid index is built and utilized to embed the Clipping Polygon (CP) into the Clipped TIN (CTIN) by interpolating the CP’s vertices’ elevation and solving the intersections of the CP and the CTIN. After that, the topology of the triangles situated within (outside of) the CP is reconstructed, and then the boundary polygon of those triangles is obtained based on the reconstructed topology. Finally, a new boundary TIN between the CP and the boundary polygon of the triangles situated within (outside of) the CP is generated by applying the one-time edge-prior constrained Delaunay triangulation (CDT) growth algorithm, and the TIN to be clipped out is separated from the CTIN by topology modification. At that point, CTIN clipping is accomplished with the local details remaining. The algorithm has been programmed in C# and.NET. Additionally, it is also applied to the opencast coal mine digital mining design practice, and it is robust and highly efficient.

## 1. Introduction

Constructing a digital elevation model (DEM) of geological objects is the basis of digital mining design in opencast coal mines [1]. The DEM is described by different types of structures, such as regular square grids (gridded DEM, GDEM), triangulated irregular networks (TIN) and contour-based structures. As the TIN has some advantages, such as perfect topology, high accuracy and variable resolution, it has been considered to be better than the GDEM [2]. Therefore, the TIN is usually a preferred DEM structure to describe the stratiform geological objects of coal deposits.

In the process the opencast coal mine digital mining design based on a 3D geological model, it is inevitable to clip the DEM described by the TIN of the geological object. Therefore, the TIN clipping algorithm has become the key algorithm in the digital mining design of opencast coal mines, and it can be applied in practice, such as for partially updating the deposit stratum DEM [3,4], rock and coal volume computing [5], the mining design scheme previewing or the playback of the mining and stripping process based on virtual reality (VR) technology [6], partial geological TIN generation for digital twin (DT) technology application [7,8], and other applications related to the digitalization and intellectualization of coal mines [9].

According to different types of clipping objects, TIN clipping can be divided into surface/surface clipping and curve/surface clipping. As spatial curves can be approximated by polylines, curve/surface clipping is usually replaced by polyline/surface clipping [10]. Additionally, some surface/surface clipping algorithms were introduced in the collected literature. Maillot [11] proposed an algorithm of clipping the triangles strip by the plane derived from the Sutherland-Hodgman polygon clipping algorithm [12]. Lindenbeck et al. [13] implemented a TRICUT program using the RAPID (Robust and Accurate Polygon Interference Detection) [14] and TRIANGLE libraries, and the program realized mutual clipping of the TIN by computing TINs’ intersection lines and retriangulation of intersecting triangles. The algorithm introduced by Lindenbeck [13] has been improved by Hua et al. [15] by the surface’s collision detection with the TIN’s OBB (Oriented Bounding Box) tree, the triangles’ intersection lines solving, intersection’s coordinates normalizing and the Clipped TIN(CTIN) regenerating. Li et al. [16] improved the TIN clipping efficiency by solving the intersection lines between the TIN and the rectangle grid instead of solving the TIN intersections.

Research findings show that the surface/surface clipping algorithm is more mature than the edge/surface clipping algorithm because the spatial curve is too complex to clip the surface arbitrarily.

Zhong et al. [17] projected the TIN onto a 2D plane and then inserted the edges of the clipping polygon (CP) into the TIN using the CDT algorithm. Finally, TIN clipping is accomplished such that the extra triangles located outside of the CP are eliminated according to the edge-triangle topological relationship. A TIN clipping algorithm based on topology tracing was introduced by Yang et al. [18], utilizing the space curve on the TIN to trace by the TIN’s topology structure, thus, disconnecting the TIN from and along the curve. Huang et al. [19] proposed a triangular mesh cutting algorithm with grid topology. After obtaining the first triangle closest to the view point, all triangle sets closest to the view point and the boundary triangles are obtained according to the grid topology, and then the boundary triangles are retriangulated with edge constraints.

As the algorithm proposed by Huang et al. [19] does not mention how to maintain the accuracy of the cut triangular mesh, we do not further analyze it. In the algorithm presented by Zhong et al. [17], the CP’s edges are inserted into the CTIN, and the edge-triangle topology is used to delete the triangles outside of the CP. In the algorithm proposed by Yang et al. [18], TIN clipping is accomplished by inserting the CP vertices into the CTIN triangles, and edges or triangles are “broken” with the location relationship between the vertex and the triangles. In the end, the TIN is regenerated utilizing the topology of the TIN. In the abovementioned two algorithms, the local details of the CTIN were lost because the intersections between the CP’s edges and the edges of CTIN’s triangles have been neglected, and the CTIN was distorted and unable to maintain the accuracy of the represented object. In this paper, we propose a precise TIN clipping algorithm that can retain the local details of the CTIN. In the algorithm, a spatial grid index is built and utilized to embed the Clipping Polygon (CP) into the Clipped TIN (CTIN) by interpolating the CP’s vertices’ elevation and solving the intersections of the CP and the CTIN. After that, the topology of CTIN is constructed based on a hash function and an improved half-edge data structure. Then, the boundary polygon of those triangles located inside (outside of) the CP is obtained, and the boundary TIN between the CP and the boundary polygon is generated by applying a one-time edge-prior constrained Delaunay triangulation (CDT) growth algorithm. Finally, the TIN to be clipped out is separated from the CTIN by the topology modification. The algorithm has been programmed with C# and.NET and is applied in the digital mining design practice of the opencast coal mine.

The rest of this paper is organized as follows: Section 2 introduces the algorithm idea and its detailed implementation process, including data structure design, grid index establishing, embedding CP into CTIN, obtaining boundary edges, clipping TIN with the boundary TIN generating and CTIN separating. In Section 3, the algorithm experiments are carried out with different amounts of data; based on that, the performance of the proposed algorithm is discussed by comparing it with the other algorithms. In Section 4, the application of the algorithm in the practice of digital mining design in an opencast coal mine is introduced. Finally, the study is concluded in Section 5.

## 2. Algorithm

### 2.1 Algorithm idea

The TIN clipping algorithm’s time efficiency is affected by the number of CTIN triangles and CP vertices with the common traversal method. Thus, it is necessary to build a spatial grid index utilizing the CTIN range and length of the CTIN triangles’ edges. When the spatial grid index is built, we can map the CP’s edges and the CTIN’s triangles to the grid index cells. Based on that, the CP is quickly embedded into the CTIN by interpolating the CP’s vertices’ elevation and computing the intersection points between the CP and the CTIN’s triangles. After that, the triangles located inside (outside) of the CP are determined by the position relationship of the triangle’s vertices and the CP, and a “vertex-edge-triangle” topology of those triangles is constructed. Utilizing that topology, the boundary polygon is obtained with the “edge-triangle” adjacent relationship. Finally, a new TIN is generated between the CP and the boundary polygon, separating the TIN to be clipped from the original CTIN with the topology modification of the boundary edges and their adjacent triangles. Then, precise TIN clipping is accomplished.

### 2.2 Data structure

**Fig 1:**
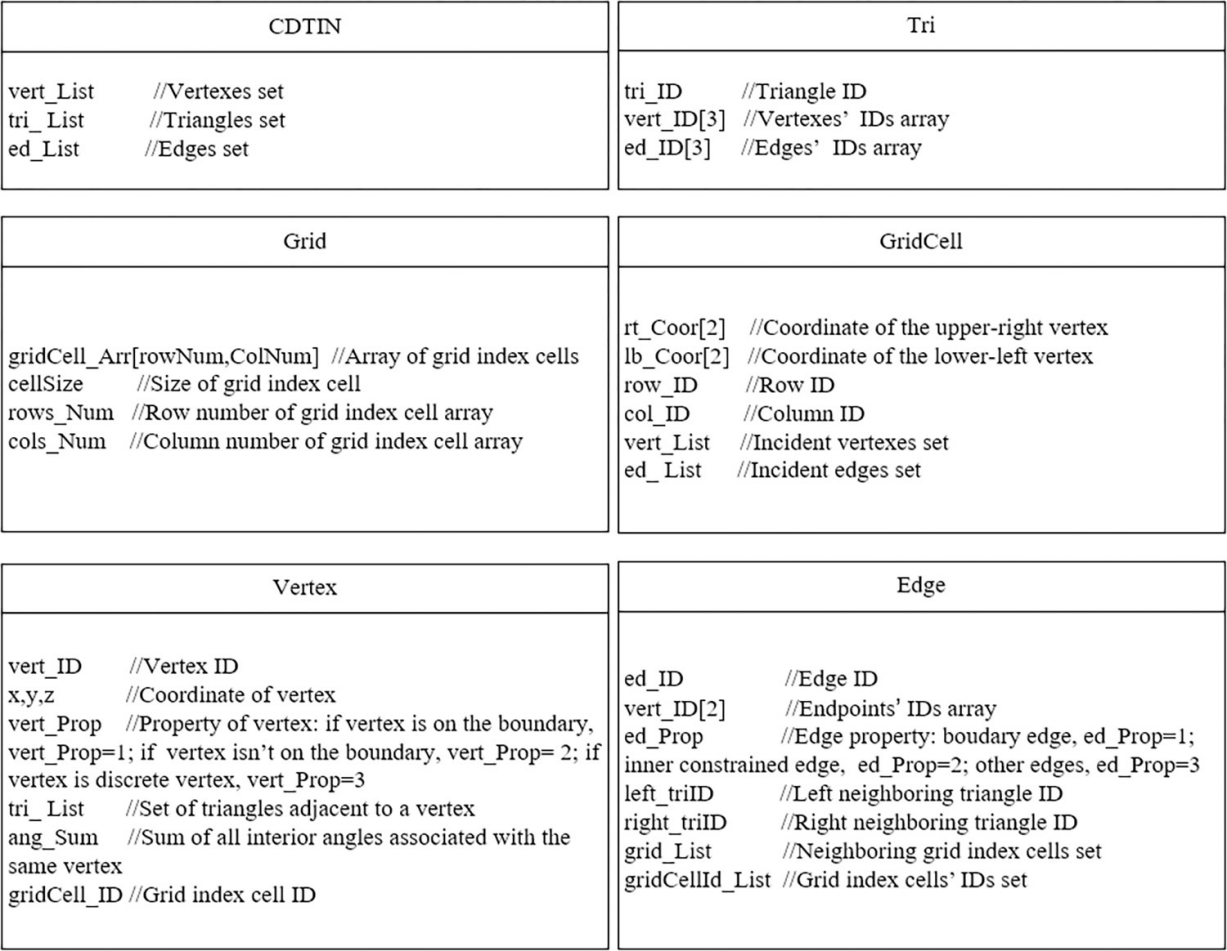
Data structure of the algorithm.

TIN, grid index, triangles, edges, and vertices are essential data objects of the algorithm Fig 1. shows the algorithm data structure defined by C# and.NET.

### 2.3 Establishing the grid index of the CTIN and the CP

To handle spatial queries effectively, a spatial index is needed. The spatial index of the CTIN and the CP can be used to rapidly locate and deal with the space object. The methods of creating a spatial index are classified as space-driven and data-driven spatial indexing methods [20]. Among the general spatial indices, the grid index is a kind of high efficiency, extreme conciseness and easily attainable index [21]. The grid index establishment procedure breaks up the minimum bounding rectangle (MBR) of the space object set into some grid cells with a given size and then maps the space object to the grid cells covered by the MBR of that object [21,22]. It is an effective means to improve the efficiency of the spatial operations.

The following steps can be executed to establish a grid index of the CTIN:

(1) Determining the MBR of the CTIN;

If the maximum and the minimum values of the *X-* and *Y*-direction coordinates of all the triangles in CTIN are signified by *X*_max_, *Y*_max_, *X*_min_, *Y*_min_, then the two vertices of the CTIN minimum enclosing rectangle main diagonal are determined as (*X*_min_,*Y*_min_), (*X*_max_,*Y*_max_).

(2) Utilizing the CTIN’s triangles quantity and their geometric properties to break up the MBR into *l*×*m* matrix grid cells;

The quantity of triangles associated with a grid index cell depends on the size of the cell, and the cell size affects the algorithm’s efficiency. While conducting experiments, the grid cell size used in this paper is 1.3 times the mean edge length of the triangles in the CTIN.

To identify each grid index cell uniquely, two integers, *i* and *j*, are defined to represent the grid index cell’s ID number in the *x-* and *y*-direction, respectively.

(3) Mapping the CTIN’s triangles to the grid cells by the position relationship between the triangle and the grid cell;

If the maximum coordinates of a triangle’s three vertices are indicated by *x*_max_ and *y*_max_ and the minimum coordinates are *x*_min_ and *y*_min_, then the scope of the grid cells involved by the triangle can be calculated as follows:

$$i:[Int(\frac{x_{\min}-X_{\min}}{cellsize})+1]\to [Int(\frac{x_{\max}-X_{\max}}{cellsize})+1]$$ (1)

$$j:[Int(\frac{y_{\min}-Y_{\min}}{cellsize})+1]\to [Int(\frac{y_{\max}-Y_{\max}}{cellsize})+1]$$ (2)

A grid index of the CP can also be established by the spatial position relationship between the polygon’s edges and the grid cells with the method mentioned above.

After creating the grid index of the CTIN and the CP, determining which triangle the point falls in and the edge-edge intersection tests only occur among those vertices, edges, and triangles mapped into the same cells when interpolating the CP’s vertices’ elevation and solving the intersections of the CP’s edges and the CTIN’s triangles.

With the grid index of the CTIN and the CP, it is not necessary to traverse all triangles of the CTIN, so the TIN clipping algorithm’s efficiency is improved.

### 2.4 Embedding the CP into the CTIN

To embed the CP into the CTIN, the CP’s nodes first need to be interpolated based on the CTIN and then the intersections of the CP’s line segments and the CTIN’s triangle edges can be resolved, finally inserting the intersections into the proper position of the CP’s node sequence.

(1) Interpolating of the CP’s vertices’ elevation

Determining which triangle the polygon’s vertex falls in is the first problem to interpolate the CP’s vertices elevation value based on the CTIN. The unified grid index of the CTIN and the CP has been established in Section 3.1 of this paper.

With the established grid index and the process for determining the point position with the triangles, determining which triangle the CP vertex falls in can be quickly solved by traversing the triangles that map to the same grid cell with the CP vertex.

To determine the relationship between the CP’s vertex to be interpolated and the CTIN’s triangles, a vector cross multiplication method is applied.

**Fig 2:**
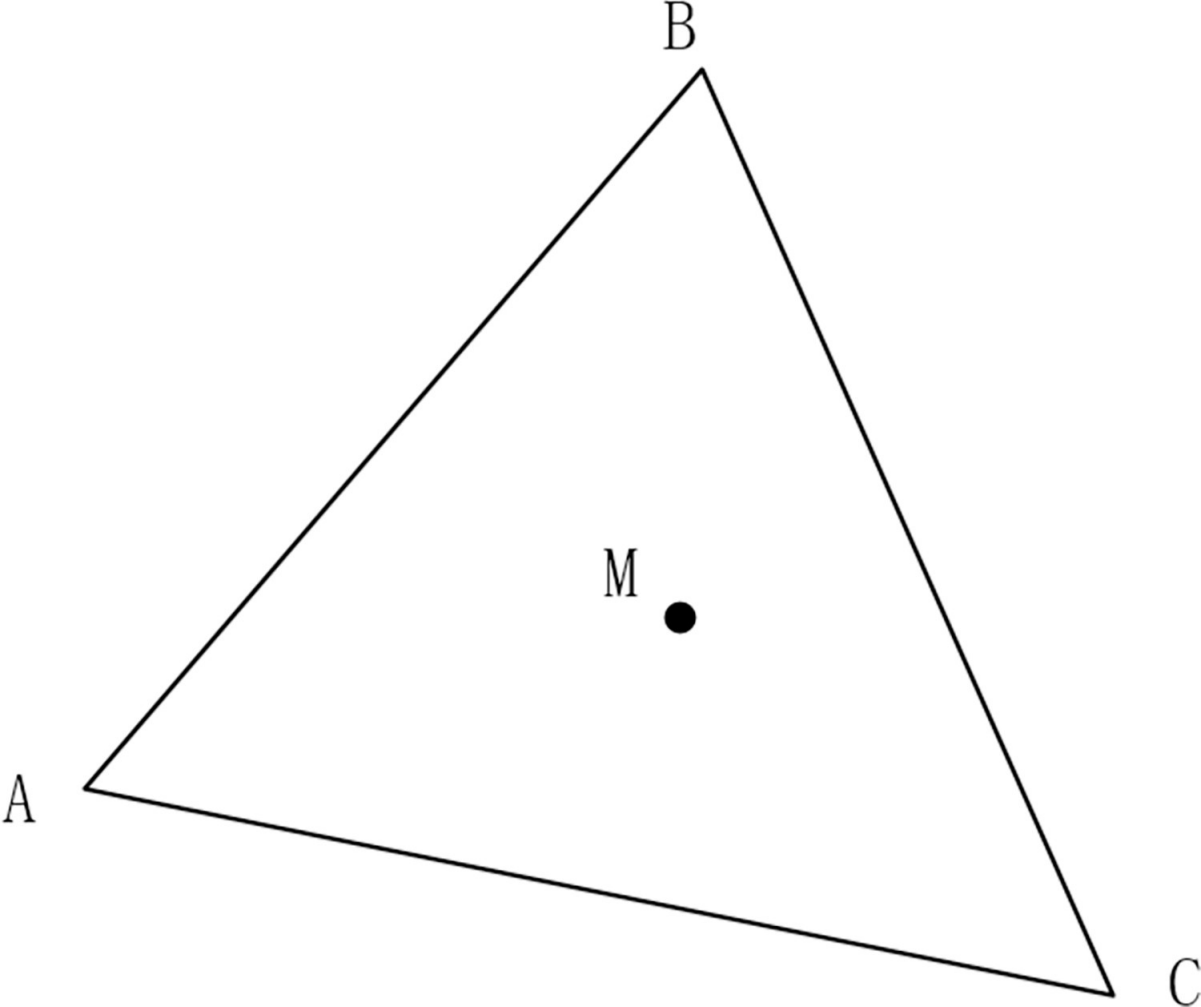
Diagram of the correlation among points and triangles.

As seen in Fig 2, the vertices of Δ*ABC* and the vertex *M* form the three vectors signified by $\vec{MA}$, $\vec{MB}$ and $\vec{MC}$, and whether the vertex *M* is inside Δ*ABC* can be determined as follows:

1) The vertex *M* is inside Δ*ABC* if any one of the conditions listed below is satisfied;

①$\vec{MA}\times \vec{MB}>0\&\vec{MB}\times \vec{MC}>0\&\vec{MC}\times \vec{MA}>0$;

②$\vec{MA}\times \vec{MB}<0\&\vec{MB}\times \vec{MC}<0\&\vec{MC}\times \vec{MA}<0$;

2) The vertex *M* is on the edge of Δ*ABC* if any one of the conditions listed below is satisfied;

① $\vec{MA}\times \vec{MB}=0$;

② $\vec{MB}\times \vec{MC}=0$;

③ $\vec{MC}\times \vec{MA}=0$;

3) The vertex *M* is outside of Δ*ABC* if none of the above conditions is satisfied.

If the vertex is inside of the triangle, its elevation can be solved with the equation of the plane defined by the triangle’s vertices.

If the vertices coordinate of Δ*ABC* are signified by (*x*_A_, *y*_A_, *z*_A_), (*x*_B_, *y*_B_, *z*_B_) and (*x*_C_, *y*_C_, *z*_C_), then the plane vector $\vec{n}$ will be defined by the following equations:

$$\{\begin{array}{l} \vec{n}=a\vec{i}+b\vec{j}+c\vec{k}\\ a=(y_{B}-y_{A})(z_{C}-z_{A})-(z_{B}-z_{A})(y_{C}-y_{A})\\ b=(z_{B}-z_{A})(x_{C}-x_{A})-(x_{B}-x_{A})(z_{C}-z_{A})\\ c=(x_{B}-x_{A})(y_{C}-y_{A})-(y_{B}-y_{A})(x_{C}-x_{A}) \end{array}$$ (3)

Then, the vertex *M* elevation signified by *z*_M_ can be interpolated as the following equation:

$$z_{M}=z_{A}-\frac{a(x_{M}-x_{A})+b(y_{M}-y_{A})}{c}$$ (4)

(2) Solving the intersections of the CP and the CTIN

Utilizing the established grid index, the CTIN’s triangles that may intersect with the CP’s component line segments are quickly obtained, after which the intersections of line segments of the CP and edges of the triangle are solved with the line segment intersection algorithm [23,24].

**Fig 3:**
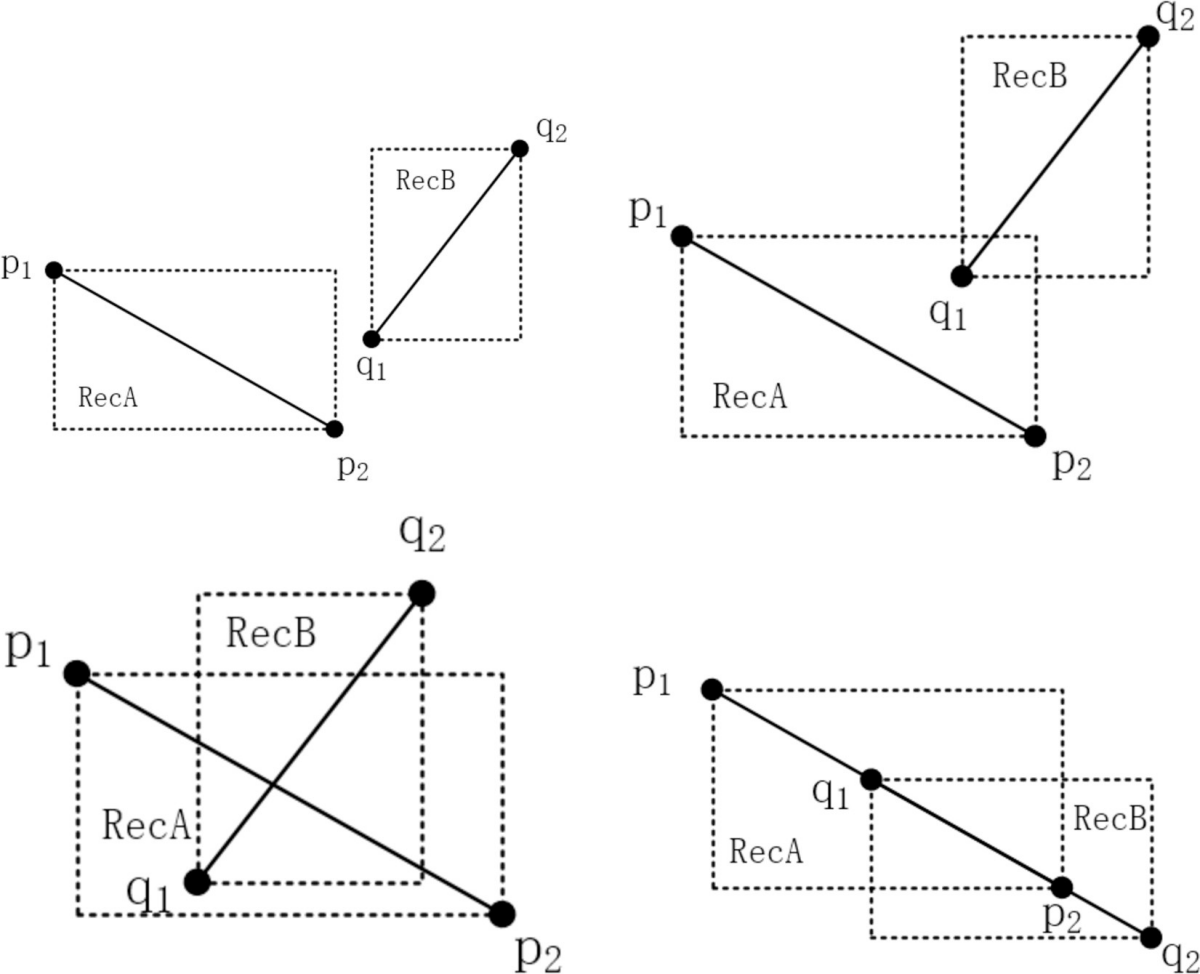
Intersection solving of two line segments.

There are three kinds of position relations between two line segments: coincidence, intersection and non-intersection. As illustrated in Fig 3, the intersection of two line segments *p1p2* and *q1q2* is resolved as:

1) Rapid rejection tests

If a rectangle with a diagonal *p1p2* and a rectangle with a diagonal *q1q2* do not overlap, then *p1p2* and *q1q2* will not intersect definitely; otherwise, *p1p2* and *q1q2* will probably intersect [25].

The following method can be used to determine whether *RecA* and *RecB* overlap:

If the inequalities RecA.minX≤RecB.maxX, RecB.minX≤RecA.maxX, RecA.minY≤RecB.maxY, and RecB.minY≤RecA.maxY are fulfilled, then we can conclude that *RecA* and *RecB* overlap; otherwise, they do not overlap.

According to Fig 3(A), *RecA* and *RecB* do not overlap, and *p1p2* and *q1q2* have no intersection; in Fig 3(B), *RecA* and *RecB* overlap, while *p1p2* and *q1q2* have no intersection; Fig 3(C) shows that *RecA* and *RecB* overlap, and *p1p2* and *q1q2* have one intersection. Therefore, the overlap of *RecA* and *RecB* will not guarantee the intersection of *p1p2* and *q1q2*.

2) Cross detection

The intersection of two line segments suggests that they definitely cross, so the following cross detection rules can be used to determine if the two line segments intersect.

① $(\vec{q_{1}p_{1}}\times \vec{q_{1}q_{2}})\cdot (\vec{q_{1}p_{2}}\times \vec{q_{1}q_{2}})<0$

$(\vec{p_{1}q_{1}}\times \vec{p_{1}p_{2}})\cdot (\vec{p_{1}q_{2}}\times \vec{p_{1}p_{2}})<0$

If the above two conditions are fulfilled, the two line segments intersect definitely.

3) Solving the intersection of the line segments

After the rapid rejection test and cross detection, the following methods are used to resolve the intersections of the intersected line segments.

As shown in Fig 3(C), designating the endpoints’ coordinates of *p1p2* and *q1q2* as *(x1*, *y1)*, *(x2*, *y2)*, *(x3*, *y3)*, and *(x4*, *y4)*, the intersection coordinates are given as follows:

$$\{\begin{array}{l} x_{0}=\frac{d_{1}}{d}\\ y_{0}=\frac{d_{2}}{d}\\ d_{1}=b_{2}(x_{2}-x_{1})-b_{1}(x_{4}-x_{3})\\ d_{2}=b_{2}(y_{2}-y_{1})-b_{1}(y_{4}-y_{3})\\ d=(x_{2}-x_{1})(y_{4}-y_{3})-(x_{4}-x_{3})(y_{2}-y_{1})\\ b_{1}=(y_{2}-y_{1})x_{1}+(x_{1}-x_{2})y_{1}\\ b_{2}=(y_{4}-y_{3})x_{3}+(x_{3}-x_{4})y_{3} \end{array}$$ (5)

After calculating the intersection’s plane coordinates of the CP’s line segments and the CTIN’s triangles, the intersection elevation can be resolved by the linear interpolation method [23,24].

After solving the intersection of the CP and the CTIN, when inserting the intersection into the CP’s vertices sequence by the distance method [23,24], a new CP is generated. To date, the CP has been embedded into the CTIN.

### 2.5 Obtaining the boundary edges of the TIN to be clipped off

After embedding the CP into the CTIN, the next step is to obtain the boundary edges of the triangles, which are inside or outside the CP.

#### 2.5.1 Finding the triangles inside/outside of the CP

**Fig 4:**
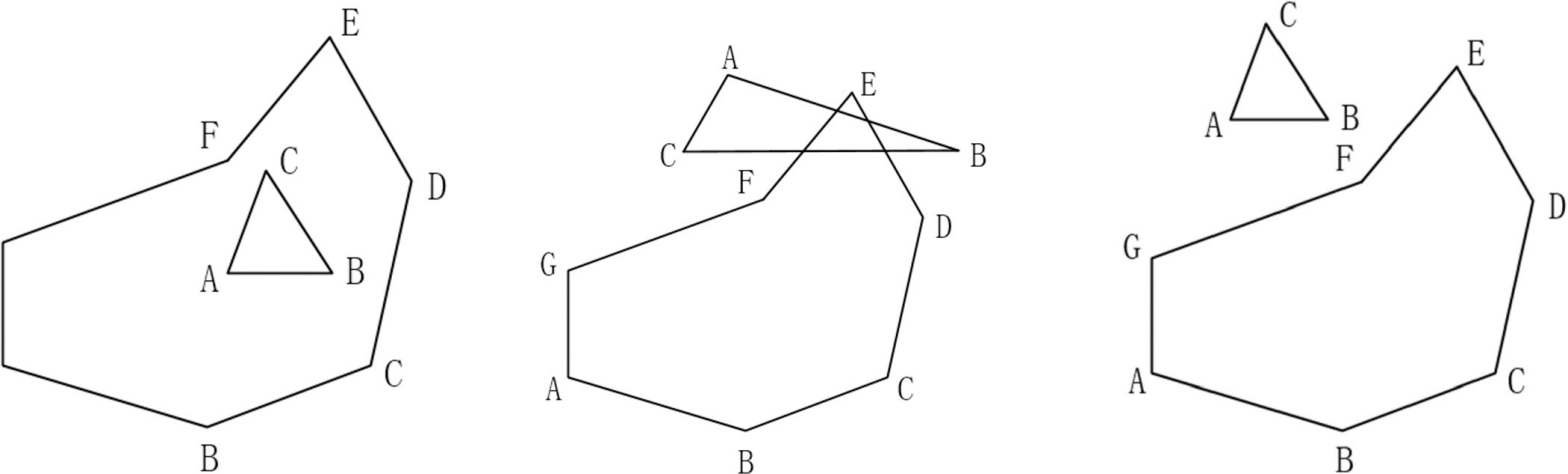
Location relationship of the triangle and the CP.

The main stage for finding the triangles inside (outside) the CP is to obtain the location relationship (inside, intersecting, outside, as represented in Fig 4) between the CTIN triangles and the CP.

If all of the triangle’s vertices lie inside the CP, the triangle definitely lies inside the CP (see Fig 4(A)); sometimes when all of the triangle’s vertices lie outside the CP, the triangle is not situated outside the CP, while it may intersect with the CP (as demonstrated in Fig 4(B)). At this time, a further intersection solving of the triangle and the CP is necessary to conclude whether the triangle lies outside the CP.

**Fig 5:**
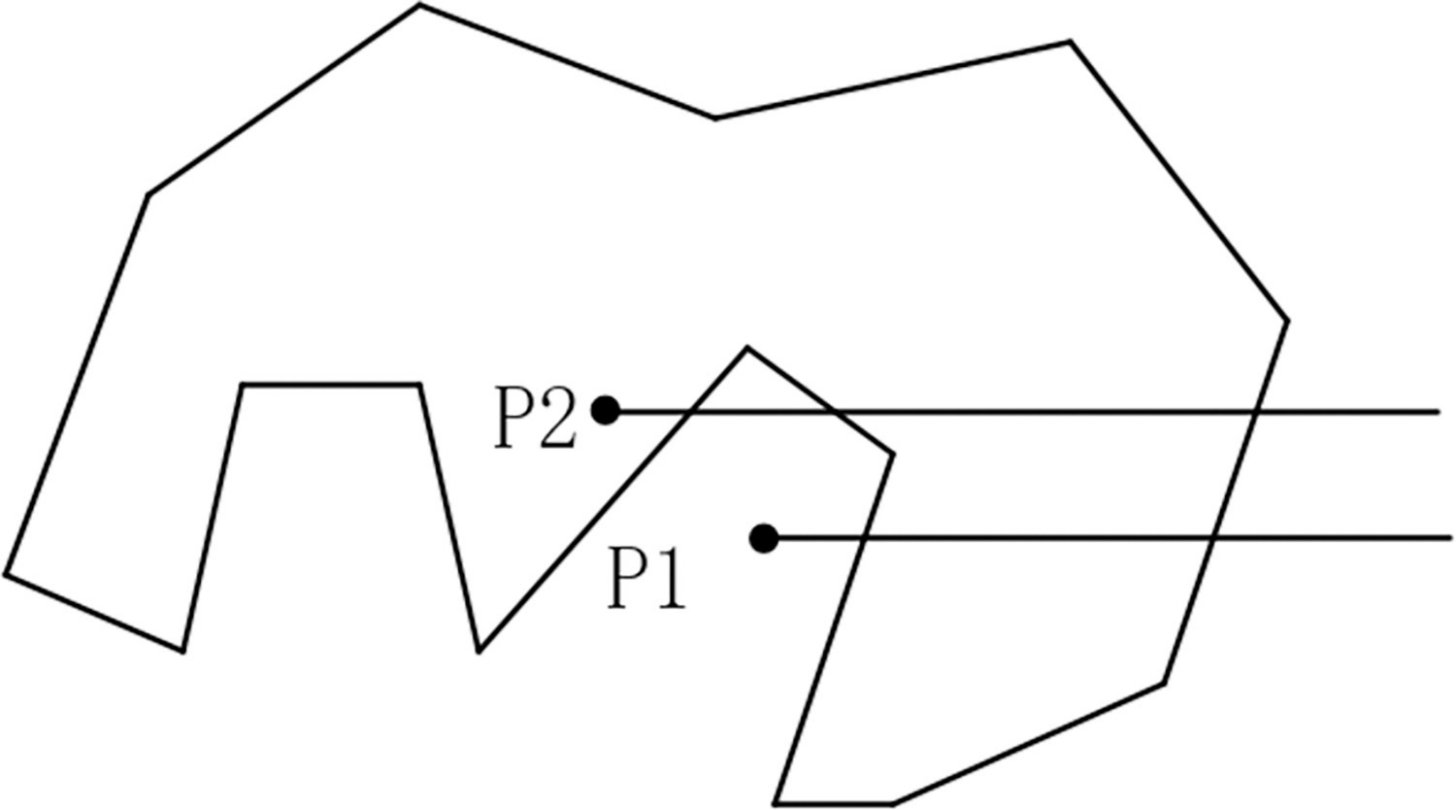
Diagram of the location relationship between points and polygons.

The grid index and the line segment intersection algorithm (mentioned in Section 3.2 of this article) can be used to determine whether a triangle intersects with CP. The position relationship (inside, outside, on edge) between the point and the polygon can be concluded by the improved ray method [26], and the procedures are as follows:

1. If the point lies outside the MBR of the polygon, the point lies outside the polygon definitely; otherwise, proceed to the next step;
2. The equations of the polygon’s edges (line segments) are used to obtain whether the point is on the edge of the polygons (here, the triangle vertex falling on the polygon’s edge is the same as the triangle intersecting with the polygon);
3. If the point is not located on the polygon’s edge, solving the intersections of the polygon and the ray emitting from the point and counting the number of intersections is necessary. This means that the point lies inside the polygon if the number of intersections is odd. Otherwise, the point lies outside of the polygon (Fig 5).

The method mentioned above can be used to obtain the position relationship of the point and the polygon regardless of whether the polygon is a convex polygon or a concave one.

The line segment intersection test algorithm described in Section 3.2 can be used to determine whether the CP and the triangle intersect.

With the process of the position relationship determination and the intersection solving among the triangles and the CP, the triangles that lie inside (outside) the CP can be found.

#### 2.5.2 Reconstructing the topology of the triangles

To obtain the boundary edges of the triangles that lie inside (outside) of the CP, the topology of those triangles with the edge-edge and edge-triangle adjacent relationship must be reconstructed.

Vertices’ aggregation and duplicate edge merging are the two main processes in reconstructing the TIN topology. The Hash function to calculate the vertex hash address and an improved half-edge data structure used to create an index table of incident half-edges for every vertex are applied in the process of vertex aggregation and duplicate edge merging. If the hash address of the vertex has a “conflict,” the list combined with the AVL tree is used to aggregate the vertex. When the vertex is aggregating, an improved half-edge data structure is used to accomplish duplicate edge merging. Thus, the edge-edge and edge-triangle adjacent relationship is established to reconstruct the TIN topology.

**Fig 6:**
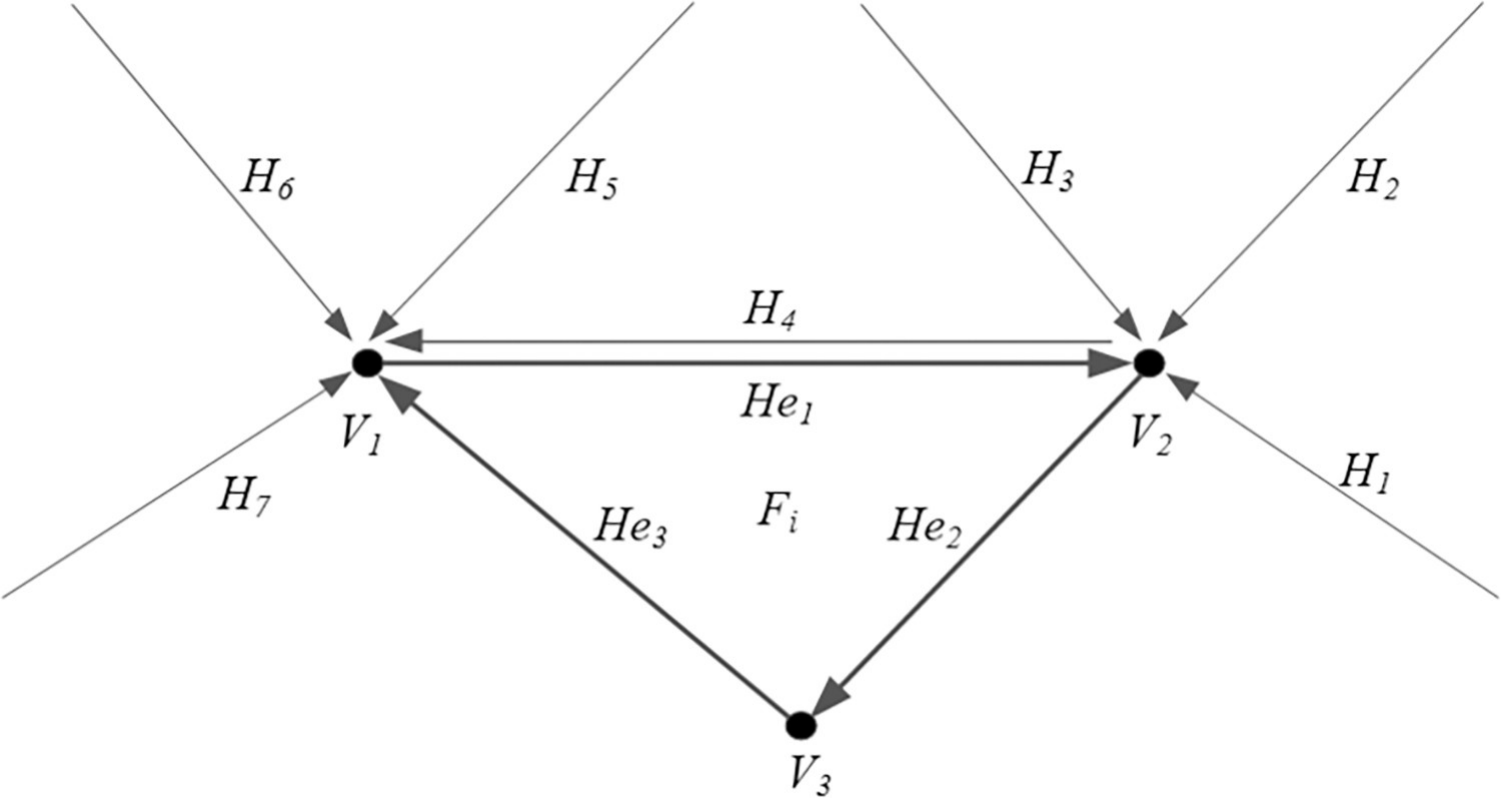
Diagram of half-edge merging.

The following steps can be performed to rebuild the TIN topology with the Hash function and the improved half-edge data structure [26]:

(1) As given in Fig 6, *F*_i_ is a triangle of the TIN;
(2) The hash addresses of the vertices of *F*_i_ can be calculated using the following Hash function:
Index=(int)((αX+βY+γZ)C+0.5)&T (6)
  where *α*, *β*, and *γ* represent the coefficients of the triangle’s vertex coordinates, and their values will directly influence the hash function’s performance. With the execution of numerous experimental studies, Jan et al. [27] concluded that *α* = 3, *β* = 5, *γ* = 7 are suitable; *C* defines the proportional coefficient. To make use of the computer’s memory as much as possible, *C* can be evaluated based on the following steps:

1) The triangle vertices’ maximum coordinates are signified by *X*_max_, *Y*_max_, *Z*_max_, then:
$$\xi_{\max}=\alpha X_{\max}+\beta Y_{\max}+\gamma Z_{\max}$$ (7)
2) *C* = *min*{*C*_1_,*C*_2_}, where $C_{1}\xi_{\max}\leq 2^{32}-1$, $C_{2}=2^{32}-2^{k}$.
  If the slot list corresponding to the vertex hash address is not empty, the coincidence determining the current vertex and the vertices in the address slot list must be done. When a coincidence occurs, the coincident vertex’s ID is assigned to the current vertex. Otherwise, the current vertex is inserted into the slot list, and num+1 is set as the current vertex ID (variable *num* denotes the number of TIN noncoincident vertices).
(3) As seen in Fig 6, the half-edge *He*_1_ of triangle *F*_i_ contains vertices *V*_1_ and *V*_2_, where both *V*_1_ and *V*_2_ have coincident vertices, thus merging *He*_1_ by finding the partner half-edge, which has the same endpoints but opposite direction with *He*_1_.
(4) To merge *He*_1_, finding the partner half-edge of *He*_1_ that has the same endpoint *V*_1_ is needed, and determining if the points are equal or not can be done by the endpoint’s ID comparison. As the TIN given in Fig 6, the half-edges with endpoints *V*_1_ are *H*_4_, *H*_5_, *H*_6_, *and H*_*7*_. With the partner half-edge rule, it is obvious that *H*_4_ is the partner half-edge of *He*_1_;
(5) We update the half-edge table with the related vertices as the endpoints. In Fig 6, the half-edge *H*_4_ is deleted from the half-edge table with the endpoint *V*_1_ (a half-edge has at most one partner half-edge), and the half-edge *He*_3_ is inserted into the half-edge table with *V*_1_ as the endpoint. Meanwhile, the half-edge *He*_2_ is inserted into the half-edge table with the endpoint *V*_3_;
(6) Inserting the half-edge *He*_1_, *He*_2_, and *He*_3_ of *F*_i_ into the TIN’s half-edge set.

Traversing every triangle that lies inside (outside) of the CP on the above six steps, the topology of the triangles is reconstructed.

#### 2.5.3 Obtaining the boundary edges of the triangles

Based on the reconstructed topology, the edge-triangle adjacent relationship can be obtained, and then the boundary half-edges that have only one adjacent triangle can be found. Those boundary half-edges compose the boundary polygon of the triangles that lie inside (outside) of the CP.

### 2.6 TIN clipping

#### 2.6.1 Generating the boundary TIN

When the CP is embedded into the CTIN, the topology and the boundary polygon of the triangles that lie inside (outside) of the CP are rebuilt, and the next procedure of the TIN clipping is to generate the boundary TIN between the CP and the boundary polygon of the triangles that lie inside (outside) of the CP.

A one-time edge-prior CDT (constrained Delaunay triangulation) growth algorithm [28] is applied to generate the boundary TIN. The applied algorithm first takes the edge with less than two adjacent triangles as the expanded edge and then searches the DT (Delaunay Triangle) point, which can form a DT with the current expanded edge by the minimum enclosing rectangle method. The above processes are repeated until the quantity of every edge’s adjacent triangles reaches 2; then, the boundary TIN has been generated completely.

Boundary TIN generation can also apply classical algorithms, such as the divide-conquer algorithm [29], the two-phase algorithm [30] and the sweep-line algorithm [31].

#### 2.6.2 TIN separating

The last process to complete the TIN clipping is to modify the topology between the boundary edges and their adjacent triangles that belong to the CTIN. The topology modification just needs to set the value of the boundary edges’ adjacent triangles number to 1. Then, the topological association between the new generation boundary TIN and the original CTIN is released, the TIN to be clipped off is separated from the original CTIN, and at this point, the TIN clipping is accomplished.

## 3. Experiments and discussion

### 3.1 Experiments

The proposed algorithm in this paper has been programmed in C# and.NET. To test the performance of the proposed algorithm, experiments are carried out based on different datasets.

**Fig 7:**
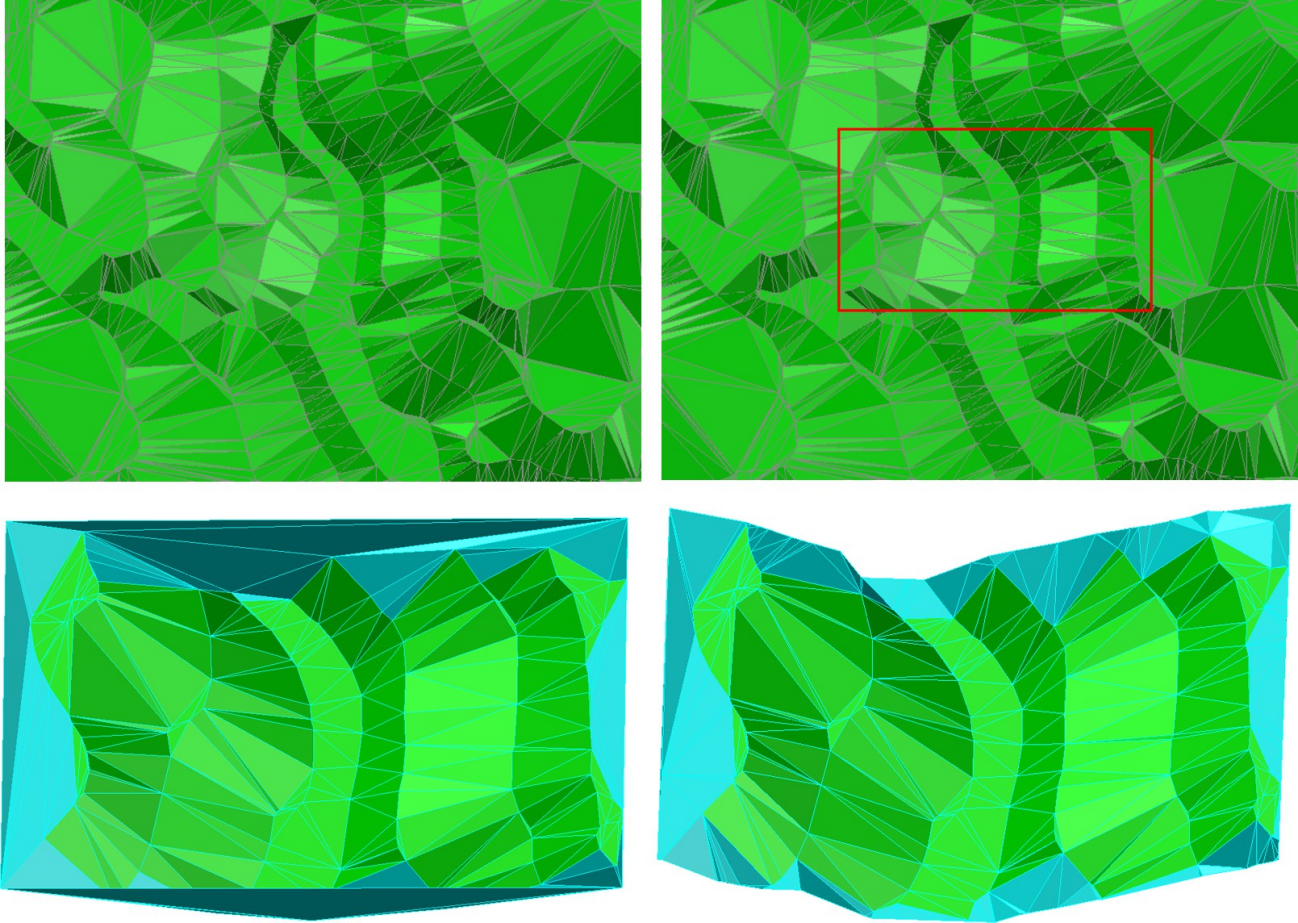
TIN clipping with a convex CP.

Fig 7 shows a simple test sample of the algorithm. Fig 7(A) presents the terrain TIN to be clipped, while the CP is shown with a red rectangle in Fig 7(B). If the local details of the terrain are neglected in the course of the CTIN being clipped, the clipping results are as displayed in Fig 7(C). Fig 7(D) shows the clipping result applying the algorithm proposed in this paper. The cyan triangles constitute the boundary TIN generated by the CP and the boundary polygon of the TIN to be clipped.

Taking Fig 7(C) in contrast with Fig 7(D), it is obvious that the TIN clipping neglecting the local terrain details results in the lack of fidelity of the terrain along with the CP, and the TIN to be clipped is inconsistent with the original terrain TIN (Fig 7(C)). However, applying the precise TIN clipping algorithm proposed in this paper to clip the TIN, with the CP’s vertices’ interpolation and intersection computing of the CP’s component line segments with the CTIN’s triangles, the terrain TIN’s local features are completely preserved (Fig 7(D)).

**Fig 8:**
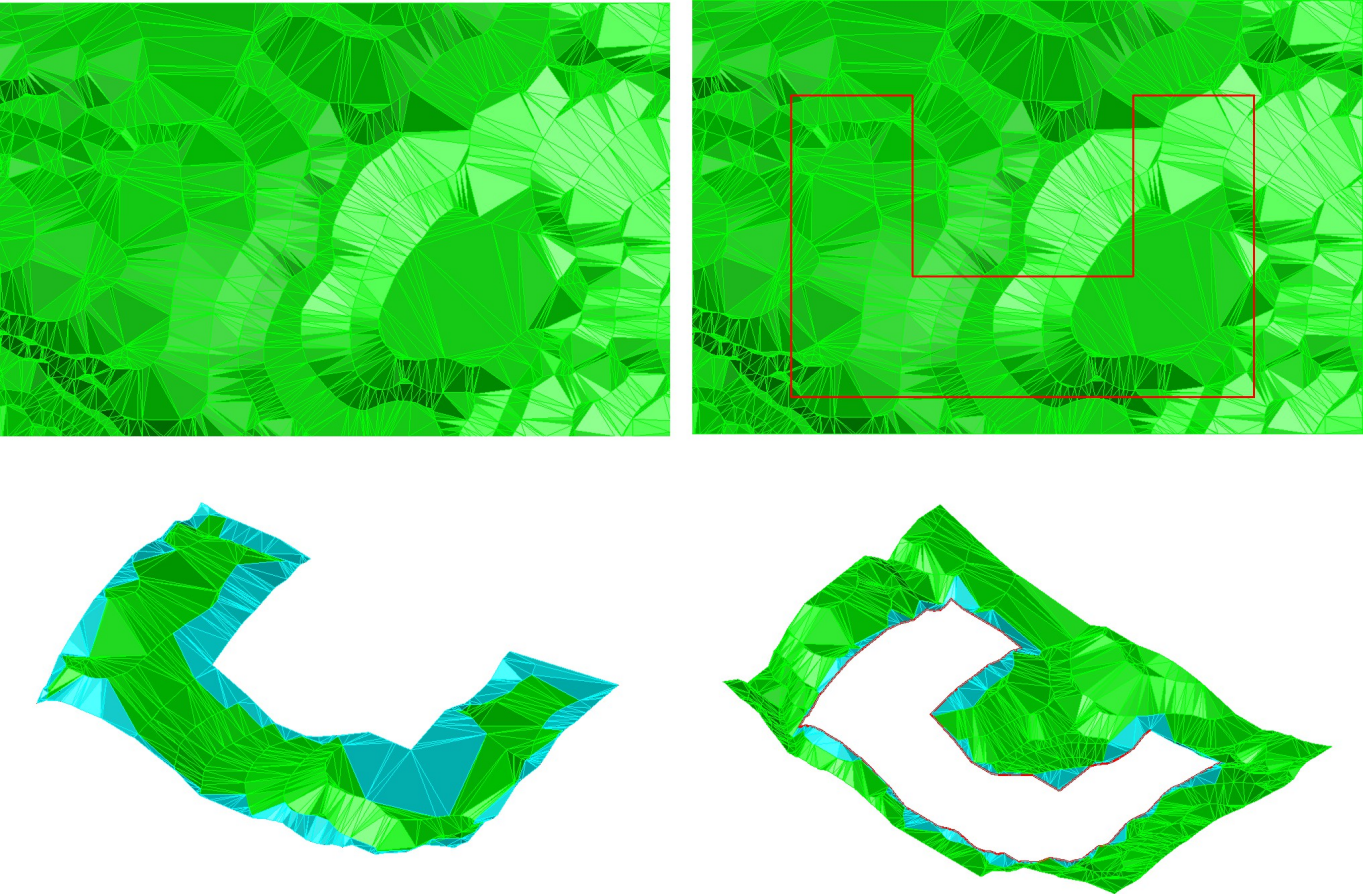
TIN clipping with a concave CP.

Fig 8 shows an experimental result of TIN clipping with a concave polygon. Fig 8(C) shows the clipping result of the remaining triangles inside the CP. The cyan triangles constitute the boundary TIN between the convex CP and the boundary of the CTIN inside the CP. Fig 8(D) displays the clipping result of the remaining triangles outside of the CP. Even if the CP is a typical concave polygon, the local details of the CTIN are well-reserved.

### 3.2 Discussion

In the process of CTIN clipping, the algorithm proposed in this paper calculates the intersection of the CP and the CTIN and reconstructs the boundary TIN of the CP, which completely retains the morphological characteristics of the CP and the CTIN along the trace of the CP, and the CP is clipped precisely while considering the local details. Algorithm experiments have proved that whether the CP is convex or concave, the algorithm proposed in this paper can achieve precise clipping of the CTIN (Figs 7 and 8), which shows that the algorithm is robust.

This algorithm applies the grid index, the improved half edge data structure and the hash function to the construction of point, edge and face (triangle) spatial index of the cropped polygon, the cropped triangulation and the topology reconstruction of the cropped triangulation to improve the query and calculation efficiency of the midpoint, edge and face (triangle) objects in the cutting process to ensure that the algorithm can still have high time efficiency under the condition of a large data scale.

To test the efficiency of the algorithm proposed in this paper, 5 groups of TINs composed of different numbers of triangles are selected using the algorithm proposed in this paper and the algorithm proposed by Yang et al. [18] to clip the TIN and record the time efficiency of the two algorithms, as shown in Table 1.

**Table 1:** Time efficiency comparison.

Dataset		Running time (ms)	
Triangles number of CTIN	Vertices number of CP	Algorithm proposed in this paper	Algorithm proposed by Yang et al. [18]
844	10	394.4	713.8
	20	312.8	594.7
	50	466.4	915.5
	100	686.7	1129.9
2864	10	560.3	1068.2
	20	685.6	1278.0
	50	941.4	1722.8
	100	1345.3	2008.9
54073	10	2829.8	5833.0
	20	3527.9	5802.6
	50	6002.0	8528.2
	100	12231.5	15497.4
107495	10	4223.6	8064.9
	20	6550.9	16685.8
	50	10920.9	15823.9
	100	18721.5	35785.6
398434	10	9064.2	18766.9
	20	15187.9	25624.4
	50	22304.7	35780.0
	100	46914.4	75698.7

**Fig 9:**
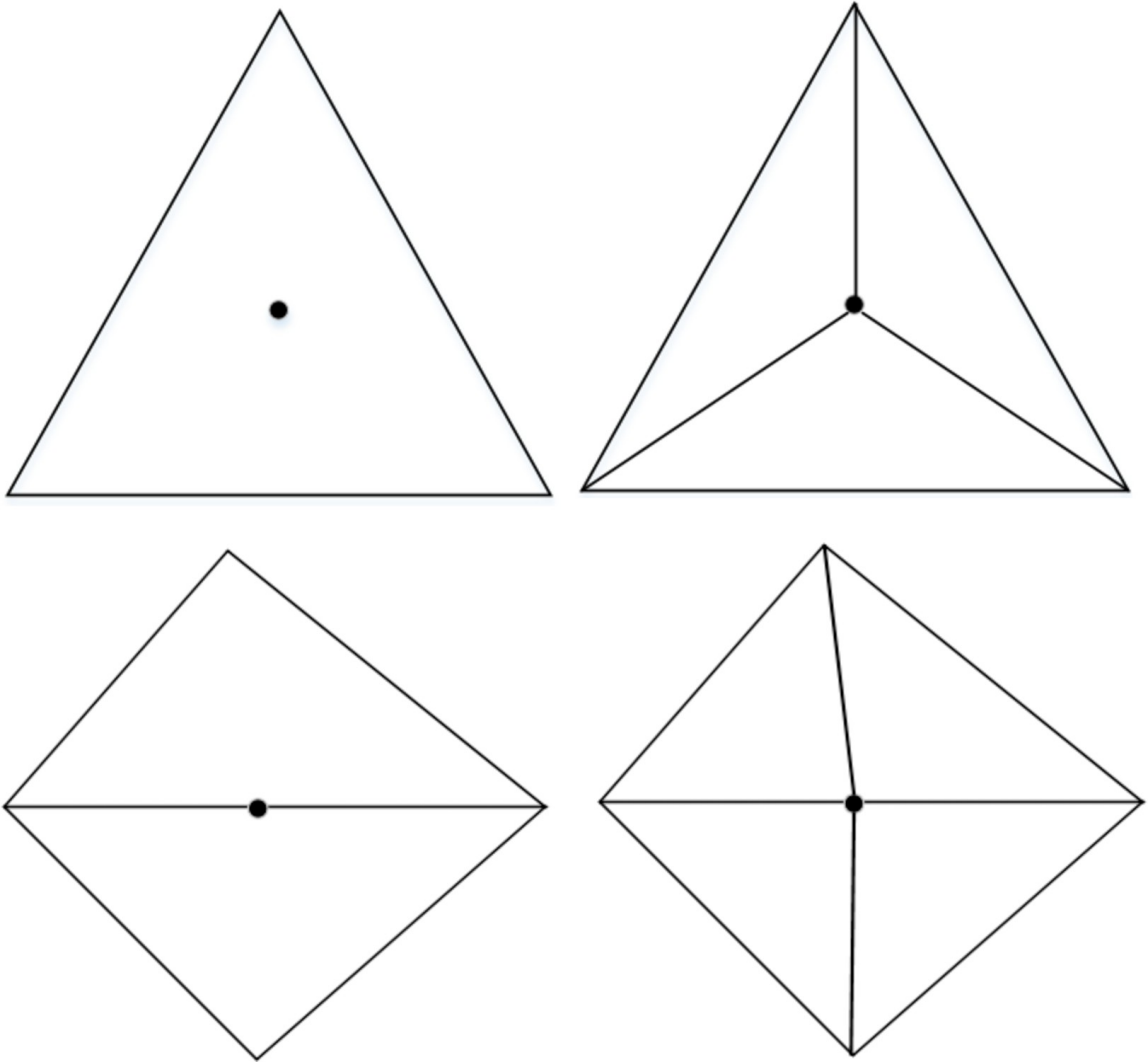
Local modification of the CTIN.

Table 1 shows that the time efficiency of the algorithm proposed in this paper is better than that proposed by Yang et al. [18]. The main reason for the analysis is that when applying the algorithm proposed by Yang et al. [18] to clip the CTIN, the CTIN is locally modified, split and reconstructed according to the position relationship between the vertex and the triangle (Fig 9). Because subsequent TIN separation is required to complete the TIN clipping, the topology of the CTIN must be updated when locally modifying the CTIN, i.e., vertex aggregation and duplicate edge merging are performed again for the newly added points, edges and faces (triangles). When the number of CP vertices and triangles of the CTIN is large, the topology reconstruction of the TIN takes a great deal of time, which leads to a decline in time efficiency.

In the process of precise clipping of the CTIN using the algorithm proposed in this paper, when generating the boundary triangulation based on the CP and the boundary edge, a one-time edge-prior CDT (Constrained Delaunay Triangulation) growth algorithm [28] with high time efficiency is applied, and the topology of the CTIN is updated with the generation of the boundary TIN. On this basis, CTIN separation can be accomplished by simply modifying the edge attributes along the trace of the CP. Therefore, compared with the algorithm proposed by Yang et al. [18], the algorithm proposed in this paper has certain advantages in terms of time efficiency.

The limitations of this study are as follows: First, because the grid index of the CTIN is constructed based on the two-dimensional coordinates of the triangles and the edges, if there are two triangles, the *X*- and *Y*-direction coordinates of their edge vertices are equal, but the *Z*-direction coordinates are not equal, the TIN clipping algorithm in this paper treats such two triangles as the same triangle, that is, the overlapping triangles in the *Z*-direction of the CTIN cannot be distinguished by the algorithm. So, the TIN overlapping in the *Z*-direction cannot be clipped correctly in this study, particularly the closed TIN. However, because the digital mining design of the opencast coal mine described in this study is based on multilayer DEMs, the algorithm can work well in the digital mining design practice. If digital mining design is based on closed TIN models, the algorithm needs to be improved. Secondly, the algorithm in this paper achieves precise clipping of the CTIN by calculating the intersections of the CP and the CTIN and reconstructing the TIN between the CP and the boundary polygon of the triangles located inside or outside the CP, to ensure precise clipping of the CTIN, there may be long and narrow triangles in the reconstructed TIN, resulting in some triangles do not conform to the empty circumcircle criteria of Delaunay triangulation, that means the clipped TIN cannot be guaranteed to be the best in shape. To fully express the stepped topographic features of the stope and dumping site, the DEM of the opencast coal mine is usually a CDT TIN constructed with bench edges or contour lines as constraint edges, inevitably some shorter constraint edges cause long and narrow triangles in the TIN. These long and narrow triangles cannot be optimized with LOP (Local Optimization Procedure), otherwise, DEM distorts the representation of the modeling object. Therefore, the existence of long and narrow triangles in the clipped TIN that do not conform to the empty circumcircle criteria of Delaunay triangulation does not mean that TIN clipping is wrong. In summary, the limitations of this study do not affect the application of the TIN clipping algorithm proposed in this paper in the digital mining design practice of opencast coal mines based on multilayer DEMs.

The exquisite mining design of opencast coal mines presents increasingly higher requirements for the accuracy of geological models. It is a trend to build high-precision deposit geological models based on multisource, heterogeneous, and massive data. When the data of CTIN and CP are large, the TIN clipping algorithm proposed in this paper is also unable to meet the application needs in terms of time efficiency. To meet the needs of TIN clipping with massive data, we continue our study in the fast, precise TIN clipping algorithm based on parallel computing technology.

## 4. Algorithm application

The proposed precise TIN clipping algorithm has been applied in the digital mining design practice of the opencast coal mine.

**Fig 10:**
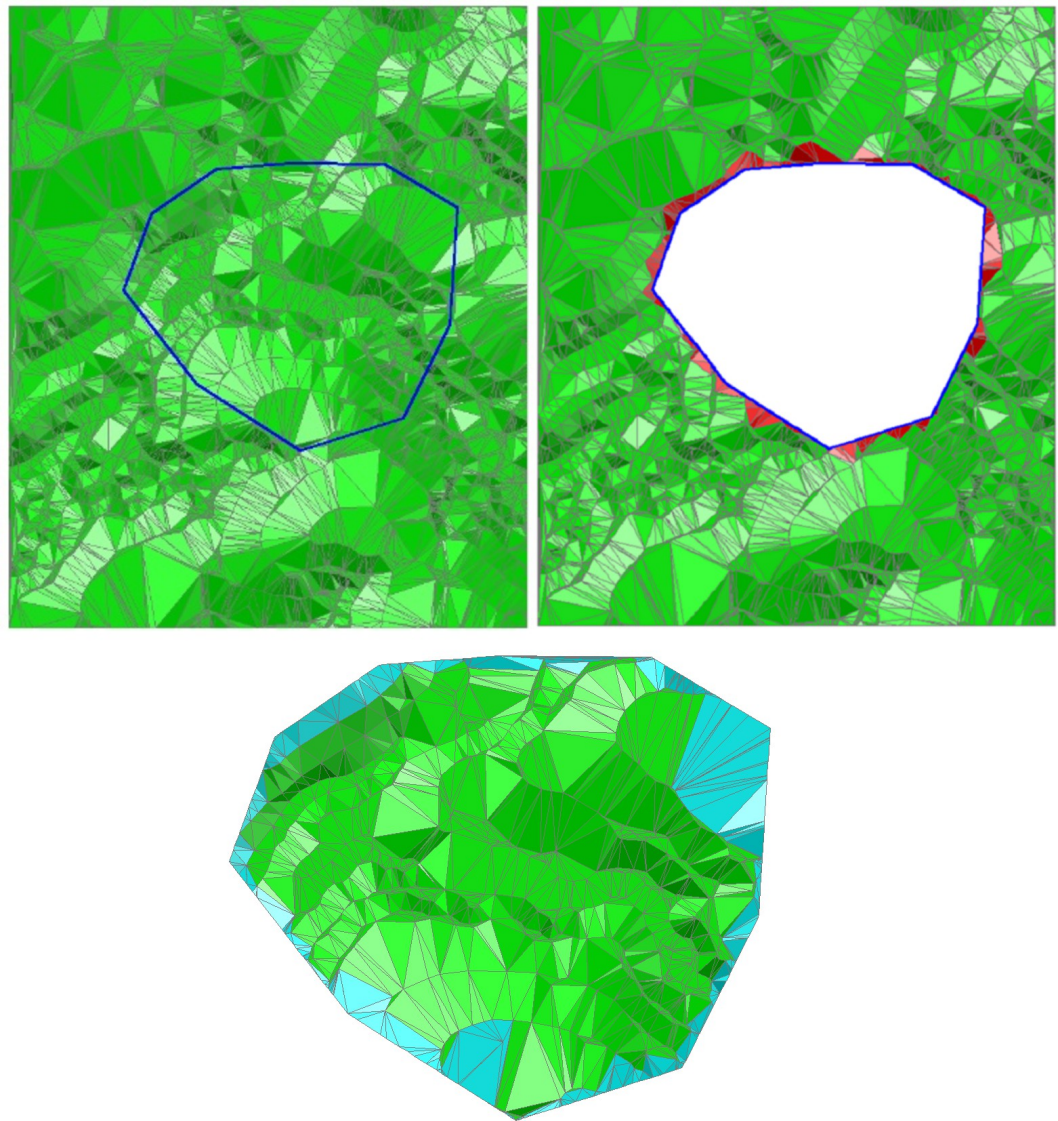
Precise clipping of the terrain TIN.

Fig 10(A) shows the original terrain DEM described by the TIN and the CP (blue polygon), and Fig 10(B) represents the TIN to be clipped precisely while the triangles lie outside of the CP remain, where the newly generated TIN based on the inserted CP and the boundary polygon of the TIN are clipped red. Fig 10(C) shows the precise clipping result, with the triangles remaining inside the CP and the boundary TIN colored cyan.

**Fig 11:**
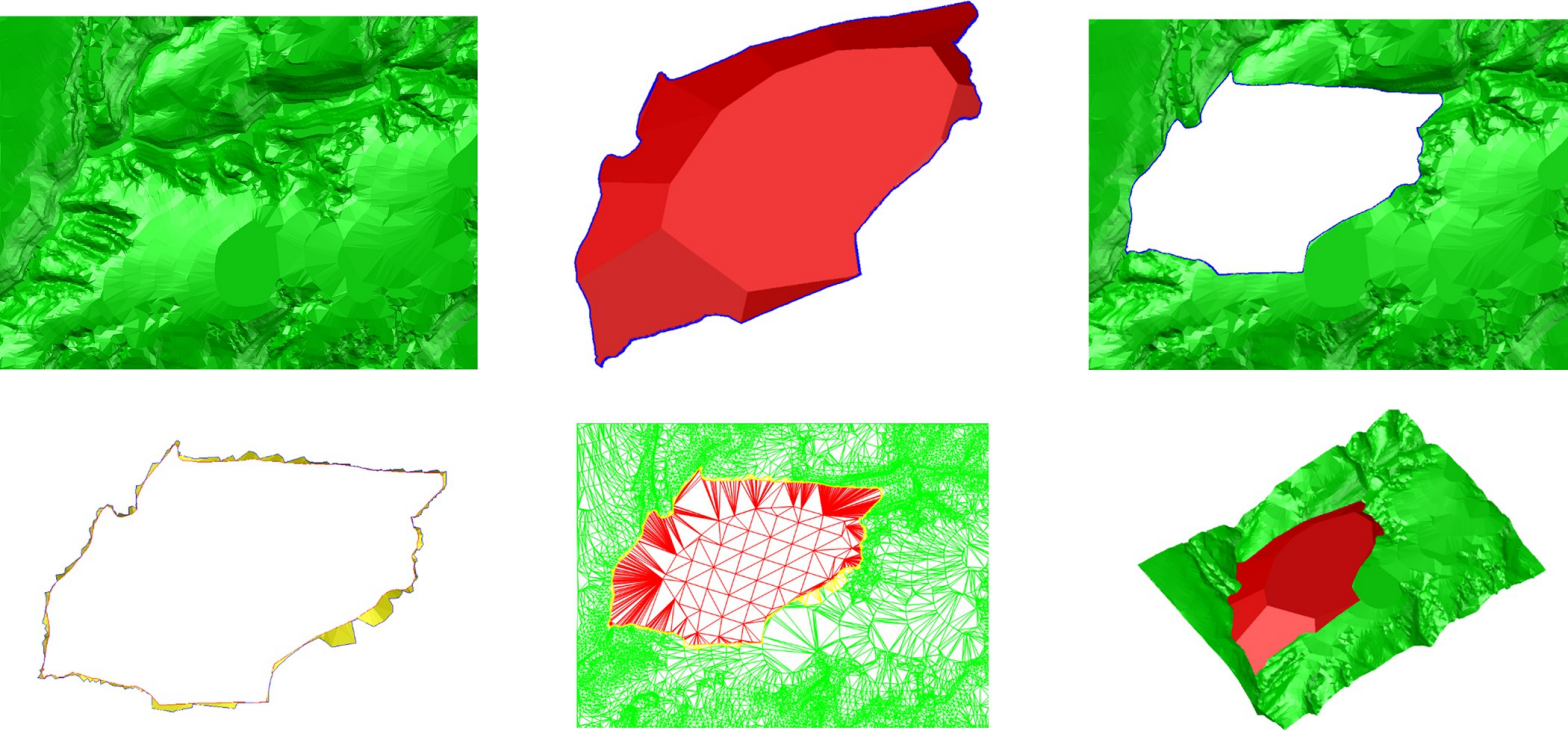
Digital design of the opencast coal mine’s dump site applying the precise TIN clipping algorithm.

Fig 11 represents the result of the TIN precise clipping algorithm applied to the opencast coal mine dump design. Taking the dump TIN’s boundary polygon as the CP (Fig 11(B)) to clip the original terrain TIN (Fig 11(A)), the precise TIN clipping is accomplished with the designed dump TIN merging with the clipped terrain TIN (Fig 11(E) is the merged TIN in the wireframe mode, and (f) is the merged TIN in rendering mode).

## 5. Conclusion

A precise TIN clipping algorithm considering the local detailed characteristics of local morphology is designed and implemented. To advance the efficiency of vertex elevation interpolation, intersection calculation, topology reconstruction and boundary TIN generation, the grid index of CTIN and the CP is established by taking 1.3 times the mean length of the CTIN triangles’ edges as the grid cell size. Additionally, a hash function and an improved half-edge data structure are applied to the vertex aggregation and half-edge merging in the process of reconstructing the TIN topology. Based on the reconstructed TIN topology, with the location relationship among the triangles and the CP, the boundary polygon of the triangles situated inside (outside) the CP is obtained. Then, the boundary TIN between the CP and the boundary polygon of the triangles that are inside (outside) the CP is generated using a one-time edge-prior CDT construction algorithm. Finally, the TIN to be clipped is separated from the original CTIN by edge-triangle adjacent relationship modification. Experiments show that whether the CP is convex or concave, the algorithm proposed in this paper can achieve precise clipping of the CTIN, and it is robust and time efficient. The algorithm has been implemented in C# and.NET and is applied in the digital mining design practice of the opencast coal mine.

## Supporting information

S1 Dataset (RAR)
